# Marine chitinase *Af*Chi: green defense management against *Colletotrichum gloeosporioides* and anthracnose

**DOI:** 10.1186/s13568-024-01786-1

**Published:** 2024-11-20

**Authors:** Rajesh K.M., Keyur Raval, Ritu Raval

**Affiliations:** 1 Department of Biotechnology, Manipal Institute of Technology, Manipal Academy of Higher Education, Manipal, Karnataka 576104 India; 2 Department of Chemical Engineering, National Institute of Technology, Surathkal, Mangalore, Karnataka 575025 India

**Keywords:** Semi synthetic colloidal chitin agar (SSCA), Marine sediment, Halophilic chitinase, *Colletotrichum gloeosporioides*, Spore germination inhibition

## Abstract

Anthracnose disease, caused by the *Colletotrichum gloeosporioides* species, affects vegetables, fruits, pulses, and cereals, leading to significant economic losses worldwide. Although many synthetic fungicides are used to control this pathogen, eco-friendly biological alternatives are gaining popularity. This study focuses on isolating and purifying chitinase ( *Af* Chi)from a marine bacterium and testing its antifungal efficacy against *C. gloeosporioides* spore germination by targeting the chitin in the fungal cell wall. The chitinase was purified from a marine bacterium *A. faecalis* from the Arabian Sea and had a molecular mass of 45 kDa and a specific activity of 84.6 U/mg. *Af* Chi worked best at 50 °C and pH 7.0 in Tris HCl buffer. Na^+^ ion was the highest cofactor, highlighting the halophilic nature of this chitinase. K^+^, Ca^2+^, Cu^2+^, Mg^2+^, Mn^2+^, and EDTA also increased activity, while Fe^3+^, Zn^2+^, Co^2+^, and Pb^2+^ decreased it. The K_m_ and V_max_ values were 1.87 µg/mL and 17.45 U/mL, respectively. Purified *Af* Chi at 10 mg/mL completely inhibited spore germination within 8 h and reduced the size of the spores.

**Supplementary Information:**

The online version contains supplementary material available at 10.1186/s13568-024-01786-1.

## Key points

- The chitinase remain stable at high temperature.
- The chitinase from *A. faecalis* shown positive result in development of natural bio-fungicide.

**Supplementary Information:**

The online version contains supplementary material available at 10.1186/s13568-024-01786-1.

## Introduction

The marine ecosystem, one of the largest and most biodiverse, is a rich source of important bioactive molecules (Ghattavi and Homaei 2023). Microbes inhabit various environments, including these marine environments which pose extreme conditions (Moreno et al. 2013). Halophilic microbes, which thrive in these extremities like high salinity, are notable extremophiles. They produce enzymes that remain stable and functional in high salt, broad pH and temperature ranges, where typical proteins would otherwise denature or aggregate (Karan et al. 2012; DasSarma and DasSarma 2015). These extremozymes are highly valuable in the biotechnology industry, with applications in bioremediation, the foods industry, biosynthetic processing, and more (Delgado-García et al. 2012; Daoud and Ben Ali 2020; Qiu et al. 2021). The bacterial halophiles produce various halophilic enzymes like xylanase, agarase, lipase, protease and chitinase (Zhang and Kim 2010; Beygmoradi and Homaei 2017; Enamala et al. 2021).

Chitinases, classified under the glycosyl hydrolase family 18 and 19, are enzymes that hydrolyse chitin, the second most abundant polysaccharide homopolymer composed of N-acetylglucosamine monomeric units (Bhattacharya et al. 2007; Yan and Fong 2015). A diverse array of organisms, such as fungi, plants, insects, animals including bacteria synthesize chitinases for various functional roles. These roles encompass nutrient acquisition, defence against phytopathogens, and morphogenesis (Aam et al. 2010; Hartl et al. 2012; Rathore and Gupta 2015). Specifically, the marine bacteria produce chitinases that play crucial roles in nutrient cycling (Souza et al. 2011) and the ecological dynamics of marine environments (Paulsen et al. 2016). Chitinase have important applications in many fields like medicine, food, biotechnology and agriculture (Gomaa 2021).

In the field of agriculture, fungal phytopathogens represent significant threats to plant and crop health, leading to decreased yields and diminished quality (Sandhu et al. 2017). A particularly concerning pathogen, *Collectotrichum gleosporides*, targeting both during field post-harvest fruits and vegetables, significantly reduces shelf life and causes substantial economic losses (Peralta-Ruiz et al. 2023). This pathogen is responsible for anthracnose disease, which is marked by the desiccation of plant tissues and wilting. *C. gleosporides* can remain dormant until post-harvest stages, at which point it induces fruit rot, exacerbating economic impacts (Fang et al. 2021). It’s devastating effects of damage are reported in many countries across the globe including India, Australia, China, United States, New Zealand and many others (Peralta-Ruiz et al. 2023). Synthetic fungicides are commonly applied at various stages of harvest to protect fruits and vegetables from anthracnose. These fungicides include thiabendazole, captan, maneb, prochloraz, benomyl, and chlorothalonil. While they reduce infection rates, their use is limited due to regulatory scrutiny in different countries (Jenny et al. 2019; Peralta-Ruiz et al. 2023). In this regard, many have alternatively explored chitinases with potential antifungal activity against phytopathogens isolated from bacteria like *Bacillus subtilis* (Senol et al. 2014; Kurniawan et al. 2022), *Bacillus licheniformis* (Ajuna et al. 2023), *Bacillus thuringiensis* (Gomaa 2012), *Bacillus cereus* (Chang et al. 2003), *Paenibacillus ehimensis* (Liu et al. 2020), *Streptomyces sp.* (Karthik et al. 2015), *Citrobacter freundii* (Meruvu and Donthireddy 2014), *Bacillus pumilus* (Gurav et al. 2017), *Paenibacillus xylanexedens* (Zhang et al. 2021), and many more. The present study explores the antifungal potential of halophilic chitinase ( *Af* Chi) against *Colletotrichum gleosporides* as a sustainable alternative to synthetic fungicides.

## Materials and methods

### Materials

All microbiological media components and analytical-grade chemicals were procured from the Hi-Media Laboratory in Mumbai, India. The acquisition of Sephadex G-100 was made by Sigma-Aldrich Inc., located in St. Louis, MO.

### Sample Collection, isolation, and screening of chitinase producing bacteria

**Fig. 1:**
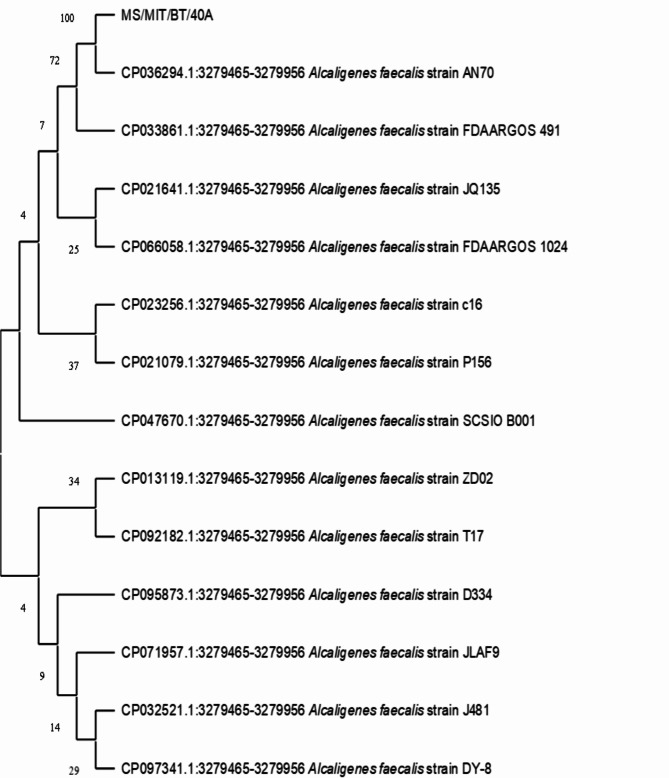
The phylogenetic tree of *Alcaligenes faecalis* was constructed from 16 S rRNA sequencing, the closest relatives and evolutionary relationships between the organisms in the tree were determined using MEGA 11 software with 1000 bootstrap replicates

A sediment sample was obtained from the Arabian Sea at a depth of 40 m using an Ekman dredge (Anas et al. 2016). The collection site was located at coordinates 12° 57′ N and 74° 47′ E (Fig. 1a). The specimen was conveyed in a sterile receptacle to the laboratory and preserved in a refrigerated chamber until it is needed for subsequent procedures. An aliquot of 100 µL from serially diluted sample solutions was spread onto semi-synthetic colloidal chitin agar (SSCA) plates using the spread plate method (Ilham et al. 2013). The semi-synthetic colloidal chitin plate consisted of the following components dissolved in distilled water NaCl-19.45 g/L, MgCl_2_-8.8 g/L, NaSO_4_− 3.24 g/L, CaCl_2_-1.8 g/L, KCl-0.55 g/ L, NaHCO_3_—0.16 g/ L, Peptone-5 g/L, Yeast extract-1 g/L, colloidal chitin—1% (w/v) and agar—2.5% (w/v). The plates were cultured for a duration of 5 days at a temperature of 37 °C. To obtain purified single colonies, the isolated bacteria were repeatedly sub-cultured on fresh colloidal chitin plates (Sasi et al. 2020; Pawaskar et al. 2021).

### Molecular identification and phylogenetic tree construction

Colonies exhibiting chitinolytic activity, indicated by the presence of a visible halo zone, were selected for further identification using 16 S rRNA sequencing. Genomic DNA was extracted using the phenol-chloroform method. The 16 S rRNA gene fragment was amplified with an Eppendorf Thermal Cycler using universal primers 27 F and 1492R. The amplified 16 S rRNA genes were compared using the NCBI BLAST similarity search tool available at https://blast.ncbi.nlm.nih.gov/Blast.cgi. Phylogenetic analysis was then conducted by aligning our sequence with closely related sequences obtained from the BLAST results through multiple sequence alignment (Subramanian et al. 2022; Ali et al. 2024). The gene sequence has been submitted as PQ218984 in NCBI. The strain has been deposited with MTCC, India with the accession number 13736.

### Production and purification of chitinase

*A.fecalis* was cultured in a semi-synthetic media and incubated at 45 °C with an agitation speed of 160 rpm in a rotary shaker for 48 h, it was then centrifuged at 10,000 rpm at 4 °C and the obtained supernatant was subjected for protein precipitation by gradual addition of crystal ammonium sulphate with slow stirring on a magnetic stirrer, with saturation level of 70% as per a reference chart. The precipitated protein was then centrifuged, and pellet was dissolved in 50mM Tris HCl, subsequently the dissolved protein was dialyzed using snakeskin membrane against 50mM TE buffer overnight at 4 °C, with three-time buffer change pH 7 (Purwanto 2016). The dialysed enzyme was put to Sephadex G-100 size exclusion chromatography column. The column was equilibrated with 20 mM Tris-HCl buffer (pH 7.0). The enzyme was eluted with 20 mM Tris-HCl buffer, and the elution was performed at a flow rate of 0.6 mL/min (Sharma et al. 2020); Mander et al. 2016). The eluted fractions were estimated spectrophotometrically at 280 nm and the protein was quantified by Lowry assay using BSA as a standard (Fu et al. 2020).

### Enzyme activity determination by schales reagent method

Chitinase activity was determined using the Schales reagent method with some minor modifications (Ferrari et al. 2014). The enzyme activity was calculated using a chitinoligosaccharides standard curve (10–100 µg/mL). One unit of chitinase activity is defined as the amount of enzyme required to release 1 µg of chitooligosaccharides generated per minute.

### Gel electrophoresis to determine chitinase activity

The molecular weight of the chitinase enzyme was determined by SDS-PAGE using a BIORAD Mini- PROTEAN^®^ Tetra cell with a 12.5% stacking gel and a 4% resolving gel. The SDS-PAGE gel was stained with Coomassie Brilliant Blue dye and documented after destaining using a gel documentation unit. The chitinase activity was analysed using the Semi-native-PAGE. The samples were prepared by mixing with 2X sample loading buffer (62.5 mM Tris-HCl pH 6.8, glycerol, and 1% bromophenol blue) in a 1:1 ratio and incubating at room temperature for 10 min (Trudel and Asselin 1990). The samples, along with a reference protein marker, were then loaded into the gel wells, a constant current of 50 V was applied until the dye front reached the bottom of the gel. Post-electrophoresis, the gel was washed in 7.5% acetic acid and 0.5% SDS for 30 min, then stained with a SYPRO Orange protein staining solution (20 µL SYPRO Orange stain in 100 mL 7.5% acetic acid) for 1 h at room temperature on a gel rocker. Excess stain was removed by rinsing the gel in fresh 7.5% acetic acid for 30 s. The fluorescent bands were visualized under UV light (Number 1999); Zhang et al. 2021).

### Effect of temperature, pH, cofactors, time and stability on chitinase activity

The effect of temperature on chitinase activity was examined using a range of temperatures from 20 to 100 °C, with increments of 10 °C (Kumar et al. 2018). Various buffers with pH values ranging from 3.0 to 10.0 were employed to investigate the impact of pH on chitinase activity. The buffers used were citrate (pH 3.0–5.0), potassium phosphate (pH 6.0–8.0), Tris-HCl (pH 7.0–9.0), and glycine-NaOH (pH 9.0 and 10.0). Further, the impact of cofactors K^+^, Na^+^, Ca^2+^, Cu^2+^, Mg^2+^, Mn^2+^, Fe^3+^, Zn^2+^, Co^2+^, Pb^2+^, and EDTA) was investigated at concentrations of 1 mM. The enzyme-substrate reaction was allowed to react in a 50 mM Tris-HCl buffer (pH 7.0) for a duration of 2 h. The Na^+^ ion was analysed at concentrations ranging from 0.5 to 3 mM. The optimal duration for chitinase activity was determined by incubating the enzyme reaction for 2, 4, 6, 12, and 24 h. The enzyme’s stability was assessed by subjecting it to 24 h of storage at room temperature (Kotb et al. 2023), followed by measuring its activity at temperatures ranging from 20 to 100 °C. The chitinase activity was analysed using the Schales reagent method.

### Chitinase kinetic measurements

To determine the kinetic parameters K_m_ and _max_ for chitinase, substrate concentrations of colloidal chitin ranging from 0.1 to 1.14 mg/mL were used. The reactions were conducted under optimal conditions identified in previous experiments. The kinetic parameters were estimated using a Lineweaver-Burk double reciprocal plot (Sonawane et al. 2016).

### Determination of fungal spore germination inhibition activity of chitinase

The inhibition of spore germination of *Colletotrichum gloeosporioides* (MTCC 4626) was investigated using purified chitinase with a concentration of 10 mg/mL. The *Colletotrichum gloeosporioides* was grown on PDA (Potato Dextrose Agar) plates at 30 ºC for 5 days. After 5 days, conidial spores were harvested from the PDA culture, and the concentration was adjusted to 1 × 10^5^ cells/mL using distilled water. A 100 µL conidial suspension containing 1 × 10^5^ cells/mL was then transferred to a 96-well plate. The germination of spores was monitored and recorded every hour using a light microscope. Subsequently, a 100µL conidial suspension containing 1 × 10^5^ cells/mL was transferred to 96 well plate grouped into 3. Group 1 control contained 4µL of distilled water and 100 µL of conidiospore suspension, likewise, Group 2 Enzyme treated comprised of 4µL enzyme (10 mg/ml) and 100 µL of conidiospore suspension, and finally Group 3 Standard fungicide comprised of Ridomil gold 10 mg/ml and 100µL of conidiospore suspension. The wells were appropriately labelled to distinguish between different treatment groups, with each group performed in triplicates. The 96-well plate was then transferred to a moisture chamber to maintain a humidity level of 30 ºC and dark condition to mimic the optimal conditions required for the germination (Muthukumar 2018; Ye et al. 2020; Gálvez Marroquín et al. 2022). After incubation number of germinated conidiospore inhibition was counted every hour using a light microscope. Conidiospore inhibition percentage is calculated using the formula.

$$ \begin{aligned} & {\text{Conidiospore inhibition percentage}} \\ & = \frac{{{\text{Number of germinated spores}}}}{{{\text{Total number of spores seeded}}}} \times 100 \\ \end{aligned} $$

### Statistical analysis

The results are presented in the mean value ± standard deviation. Each experiment was conducted three times. The results of the experiment were graphed using GraphPad Prism 8, and the statistical analysis of spore inhibition percentage was conducted using an ordinary one-way ANOVA test p value was significant (p < = 0.05). Spore size reduction was analyzed using the Fisher Least Significant Difference (LSD) test, and p value represented significant (p < = 0.05).

## Results

### Isolation, and screening of chitinase producing bacteria

The bacterial isolates were cultivated on semi-synthetic colloidal chitin agar plates (SCCA) at a temperature of 37 °C for a duration of 5 days. A total of 80 chitinolytic bacteria were observed on the plates. Among them, 17 chitinolytic bacteria with distinct morphological characteristics were further isolated based on the presence of a visible clearance zone (> 0.2 cm) (Fig. 1 supplementary) and were cultured in a semi-synthetic broth (SSCB) containing 1% colloidal chitin and incubated at temperature of 45 °C for 72 h, with an agitation speed of 160 rpm. The isolate exhibiting highest level of activity at a temperature of 45 °C was then selected for further analysis. The preliminary microscopic examinations indicated that the specimen was a rod-shaped, gram-negative non-endospore forming motile bacterium that produces amylase, ferments sugar, utilizes citrate, and does not reduce nitrate. Additionally, 16 S rRNA sequencing was conducted and partial sequences obtained were compared with reference sequences using BLAST similarity search tool (https://blast.ncbi.nlm.nih.gov/Blast.cgi) and the submitted sequence showed to have a maximum sequence homology with *Alcaligenes faecalis* and a phylogenetic tree was subsequently constructed in MEGA-11 using the neighbour-joining method for homologous family of *Alcaligenes* sp. (Fig. 1).

### Production and purification of chitinase

*A.fecalis* was cultured in a semi-synthetic media for duration of for 72 h, the obtained crude supernatant with protein concentration of 754.1 mg and specific activity of 17.65 U/mg was centrifuged at 10,000 rpm, 4 °C and further subjected to fractional precipitation using 70% saturation of crystal ammonium sulphate. which resulted in 1.7-fold increase in specific activity. Subsequently, the precipitated protein was loaded on to Sephadex G-100 size exclusion column after dialysis, the protein concentration of the obtained fractions was estimated to be 65 mg with a 4.7-fold increase in specific activity of 84.6 U/mg (Table 1) ( *Af* Chi).

**Table 1:** Summary of *Alcaligenes faecalis* chitinase purification profile

Purification step	Total protein (mg)	Total activity (U)	Specific activity (U/mg)	Yield (%)	Fold
Crude enzyme	754.1	13,310	17.65	100	1.0
Ammonium sulphate precipitation (70%)	335.72	10,484	31.23	78.7	1.7
Sephadex G-100	65	5499	84.6	41.3	4.7

### Gel electrophoresis to determine chitinase activity

**Fig. 2:**
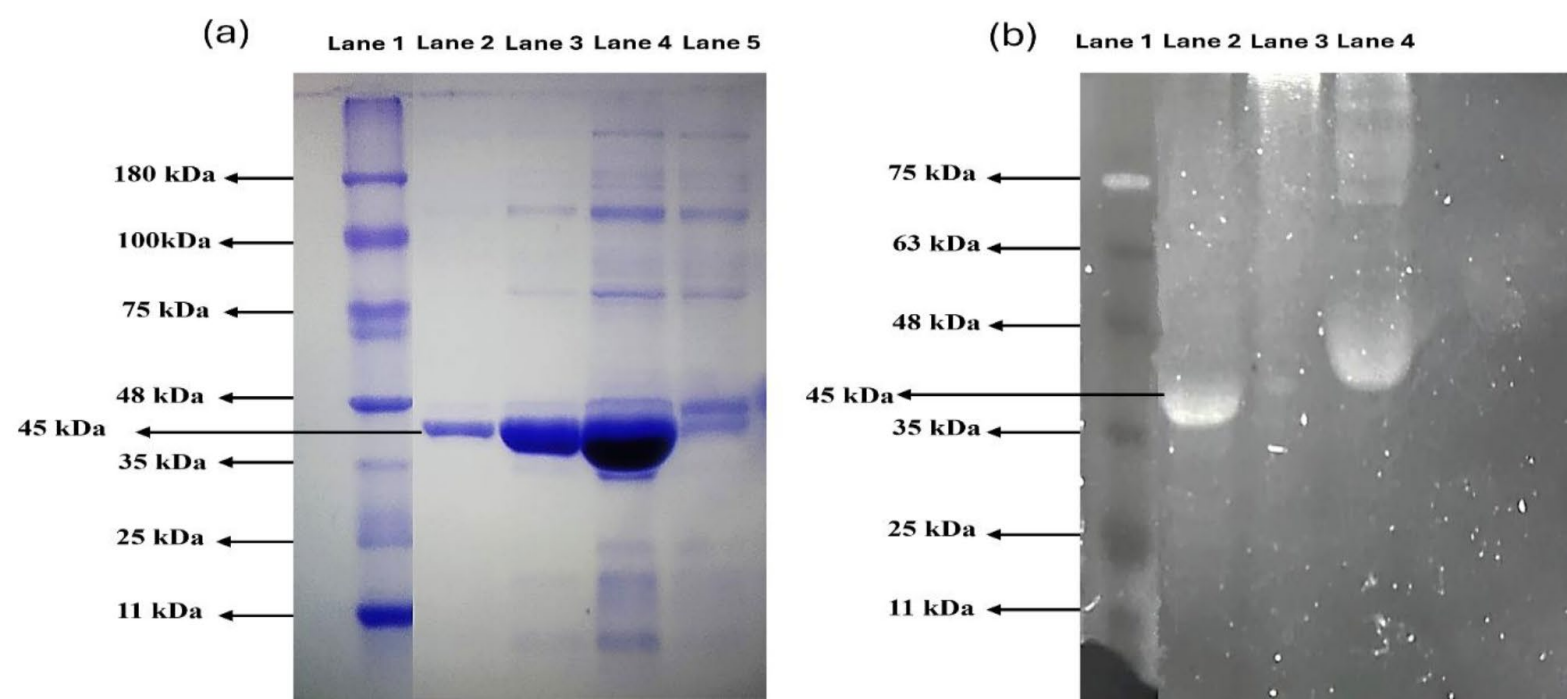
SDS-PAGE and Semi-native PAGE depicting different steps of chitinase purification from *Alcaligenes faecalis.* (**a**) SDS-PAGE gel image of protein during purification stained with coomassie brilliant blue. Lane 1, molecular marker; Lane 2, purified chitinase; Lane 3, Fraction from size exclusion chromatography; Lane 4, protein after ammonium sulphate precipitation (70%); Lane 5, crude enzyme. (**b**) Semi-native gel image of 0.1% glycol chitin stained with Sypro^®^ orange. Lane 1, molecular marker; Lane 2, purified chitinase; Lane 3, crude enzyme: Lane 4, protein after ammonium sulphate precipitation (70%)

The molecular weight of *Af* Chi was estimated using SDS-PAGE was found to be 45kD in reference to a prestained protein ladder (Fig. 2a). The Semi-native-PAGE containing glycol chitin as a substrate in the gel produced a single band at a position corresponding to 45 kDa band in SDS-PAGE which confirmed the chitinolytic activity of chitinase (Fig. 2b).

### Effect of temperature, pH, cofactors, time and stability on chitinase activity

**Fig. 3:**
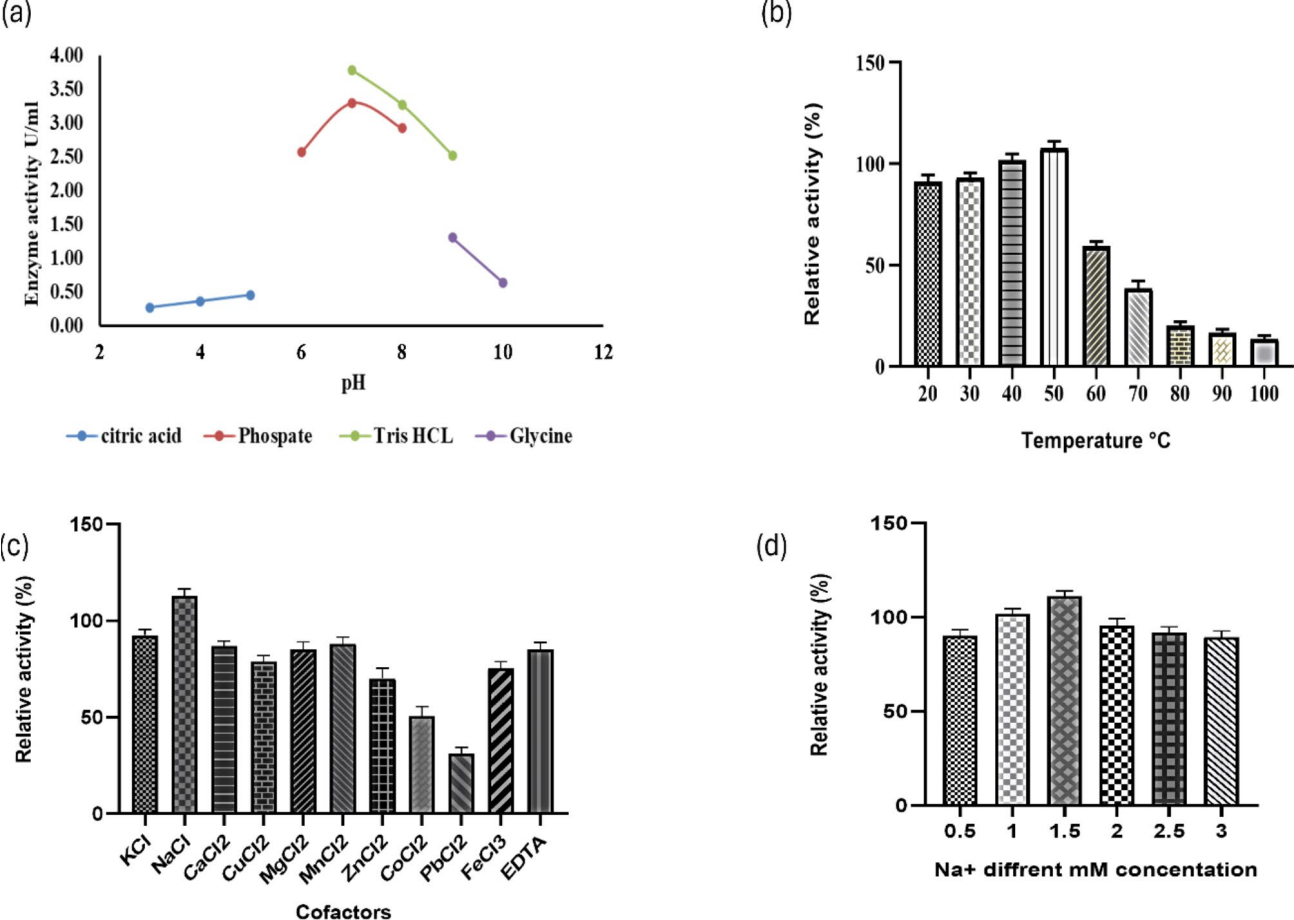
(**a**) Effect of pH on purified chitinase activity of *Alcaligenes faecalis*. The activity was assayed using citrate (3–5), potassium phosphate (6–8), tris -HCl (7–9), and glycine NaOH (9–10) buffer. (**b**) Effect of temperature on chitinase activity (20-100 °C). (**c**) Effect of different cofactors on chitinase activity. (**d**) Effect of different molar concentration (0.5-3mM) of Na^ +^ cofactor on chitinase activity

The study examined the effect of temperature and pH on *Af* Chi. The effect of the pH and buffers were examined on the purified *Af* Chi. The pH range selected for the present study was from 3 to 10. In Tris HCl buffer at pH 7.0, the enzyme exhibited its highest activity of 3.78 U/mL (Fig. 3a). An increase of pH and change of buffer beyond pH 7did decrease the activity by 10%. However further increase in the pH had a detrimental effect with 50% activity loss. The temperature range selected for the study were from 20 to 100 °C. The activity was found to increase from 20 to 50 °C. After which a sudden decrease was observed. *Af* Chi was tolerant to temperature upto 100 °C with a residual activity of 15% (Fig. 3b). The thermal stability of *Af* Chi was assessed by incubating the enzyme for 24 h at different temperature ranging from 20° to 100 °C, the enzyme was found to be stable till 50 °C, increasing or decreasing beyond this temperature showed a relative decline in enzyme activity as noted in (Fig. 2a supplementary). Among these intervals, the 2-hour incubation period exhibited the highest chitinase activity (Fig. 2b supplementary).

The inclusion of co-factors in the enzyme formulation was studied with monovalent, bivalent and trivalent metal ions. The treatment of *Af* Chi with a concentration of 1mM Na^+^ ion showed an increased relative activity of 55.01% as compared to the other cofactors like K^+^, Ca^2+^, Cu^2+^, Mg^2+^, Mn^2+^. The lowest activity was observed with the inclusion of Pb^2+^. The inclusion of EDTA did not decrease the activity further (Fig. 3c). As the highest activity was observed with Na^+^, therefore a titration of the different concentration resulted in the highest activity observed with 1.5mM with a relative activity of 111% (Fig. 3d).

### Chitinase kinetic measurements

**Fig. 4:**
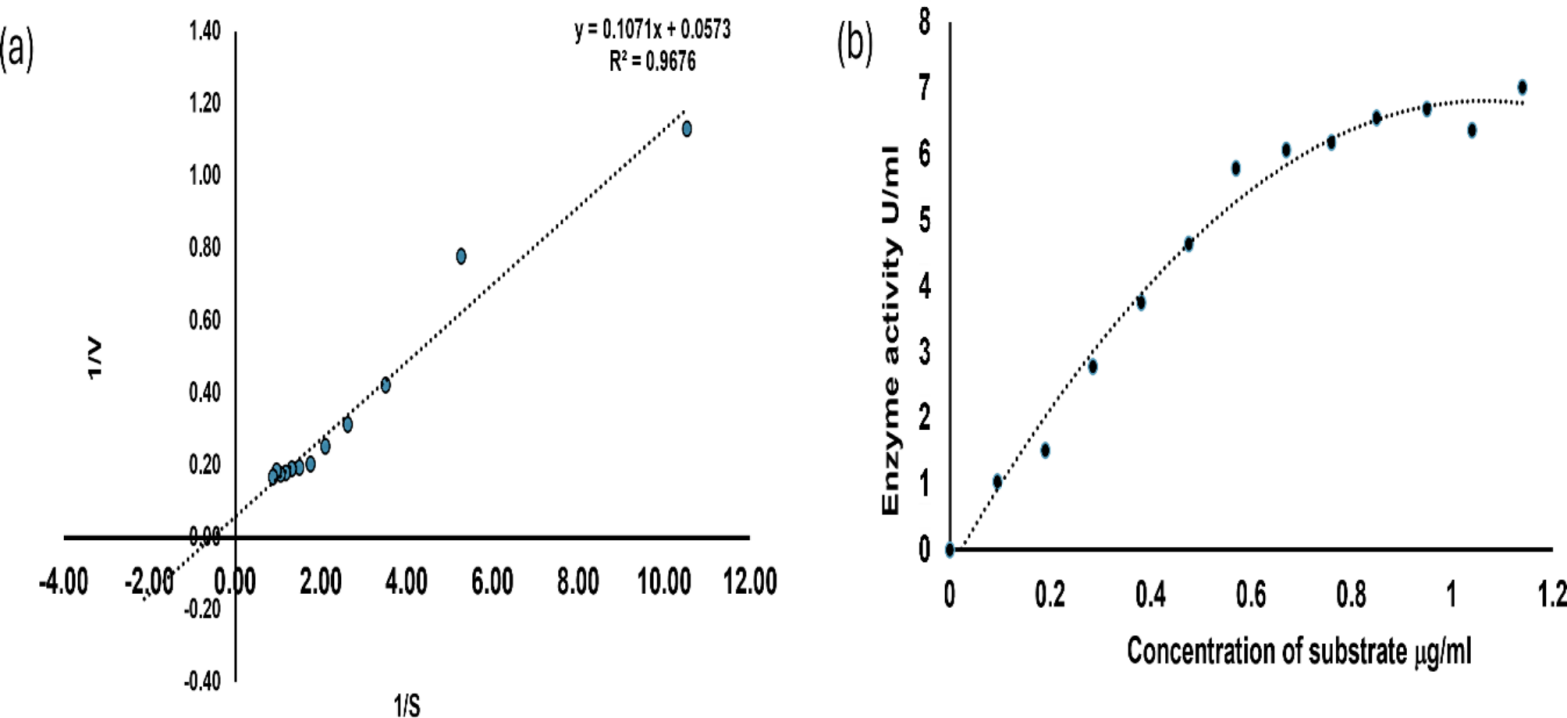
Estimation of kinetic parameters for purified chitinase using substrate concentrations of colloidal chitin ranging from 0.1 to 1.14 µg/mL. (**a**) Lineweaver-Burk plot of chitinase 1/S vs. 1/V has been used for determination of kinetic parameters. (**b**) Michaelis Menten graph. Vmax was noted to be 17.45 U/mL and Km was 1.87 µg/mL

The kinetic parameters K_m_ and V_max_ of *Af* Chi were calculated from the rate activities using colloidal chitin as the substrate, concentration ranging from 0.1 to 1.14 U/mL, performed at the optimum conditions and Vmax was noted to be 17.45 U/mL and Km was 0.187 mg/mL (Fig. 4a and b).

### Determination of fungal spore germination inhibition activity of chitinase

**Fig. 5:**
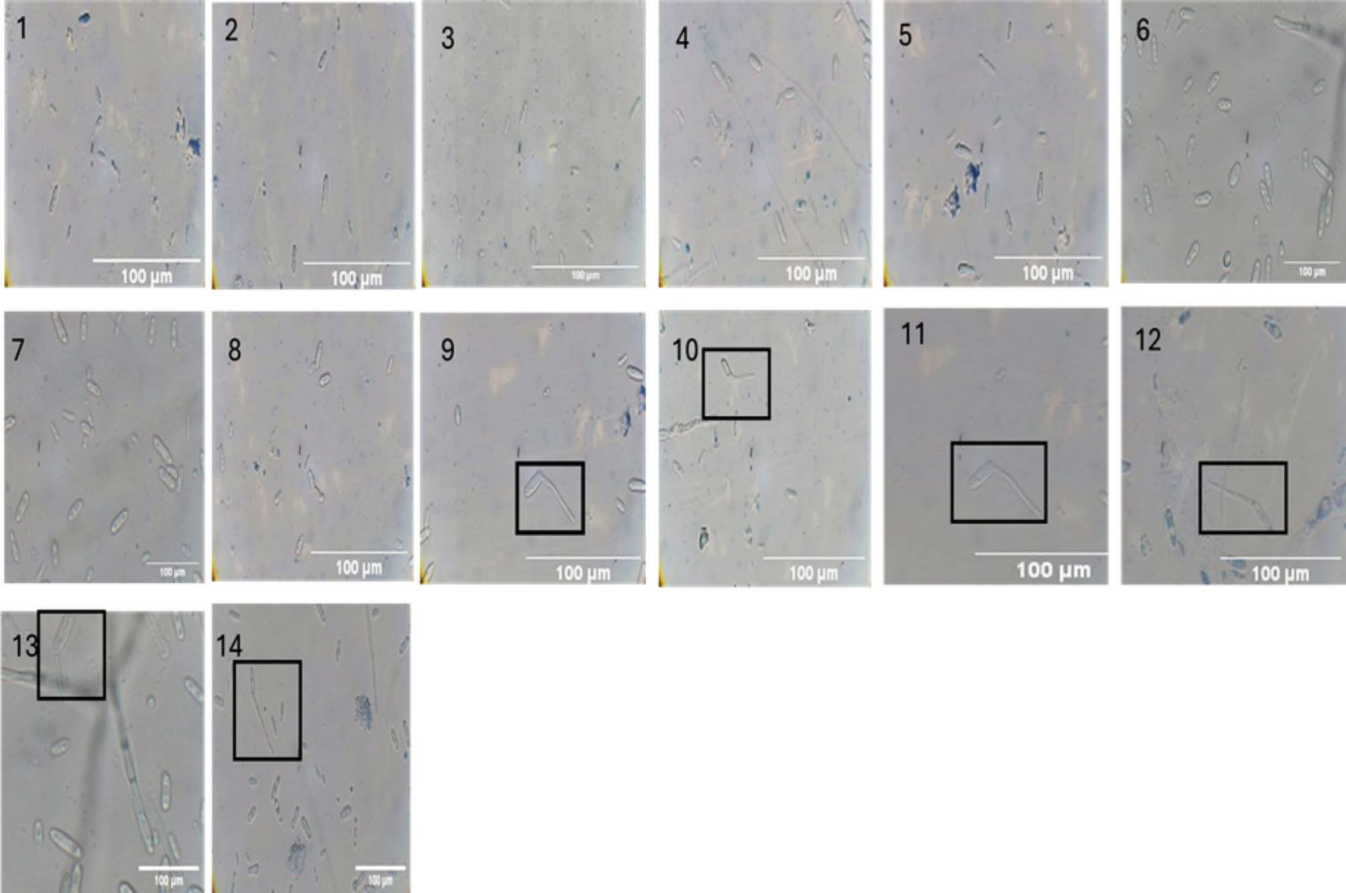
Microscopic images of hourly germination of conidiospore in group control (4 µL of water and 100 µL of conidiospore suspension 1 × 10^5^ spores) incubated for 14 h in humidity chamber germination observed from 8 h

**Fig. 6:**
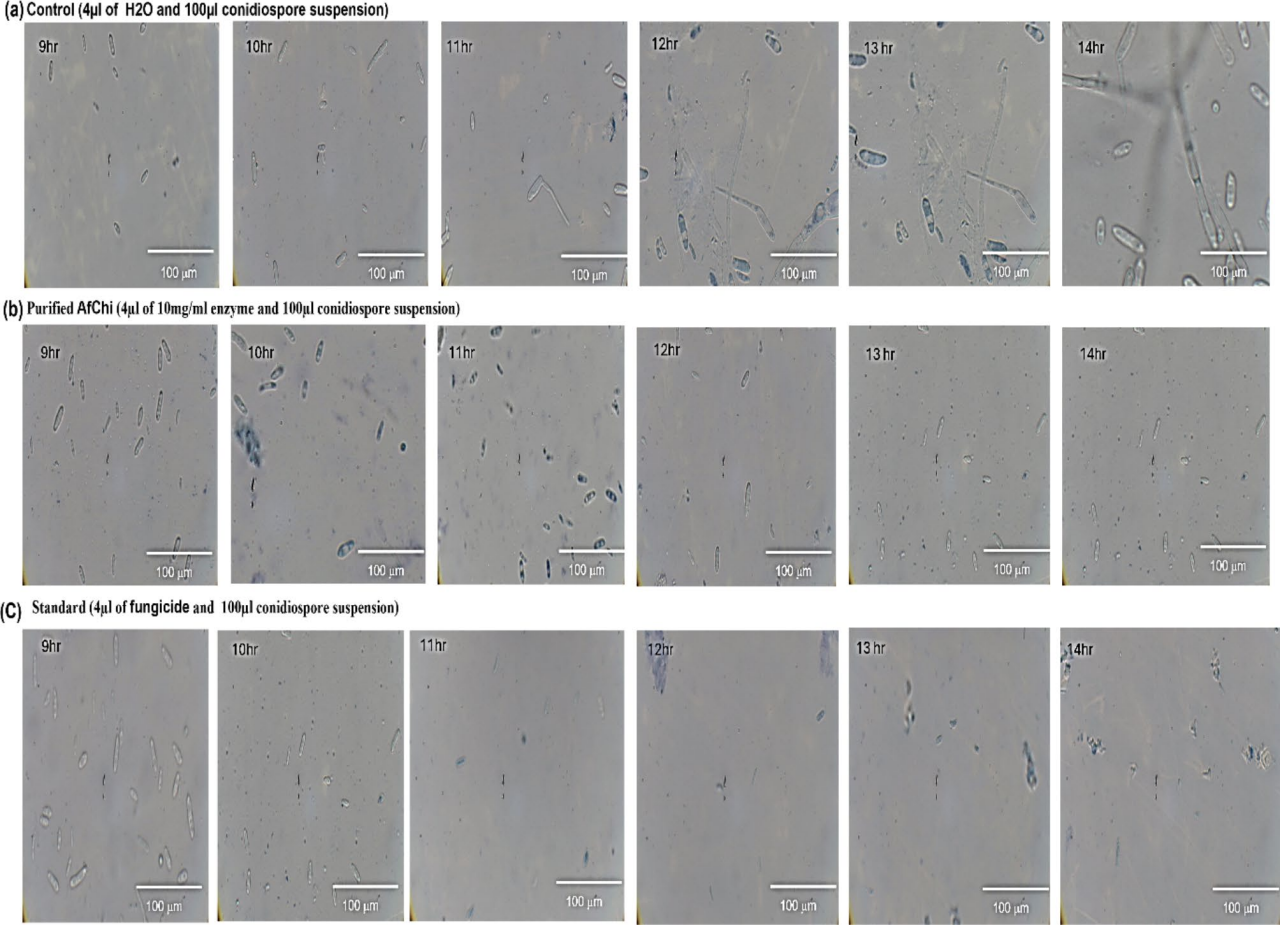
Microscopic images of study conducted inhibition of spore germination in 3 groups (**a**) Control, (**b**) Purified*Af* Chi, (**c**) Standard fungicide in 100X

A suspension of conidial spores of *Colletotrichum gloeosporioides* with a concentration of 1 × 10^5^ cells/mL, was collected from a PDA plate (Fig. 3 supplementary) and immediately introduced into a PDB culture. Notable germination was observed after 8 h, as documented using light microscopy (Fig. 5). The sprouted spores were used for a study on inhibiting spore germination, which consisted of three groups: Control Group 1, Enzyme Treated (*Af* Chi) Group 2, and Standard Fungicide Group 3 (Fig. 6). Group 2 utilized purified chitinase at a concentration of 10 mg/mL, which effectively hindered spore germination within 8 h. The cells in this group exhibited a comparatively smaller size in comparison to the control group. Furthermore, by the 11th, 12th, and 13th hours, the cells underwent further reduction. In Group 3, the application of fungicide also led to the absence of spore germination within 8 h. Furthermore, no cells were detected at 11, 12, and 13 h. On the other hand, Control Group 1 showed a significant amount of spore germination with a relatively large number of viable spores. For spore germination inhibition, between control and other two group the inhibition percentage was calculated, and ordinary one-way ANOVA test was performed p value was significant (p < = 0.05) (Fig. 4 supplementary). The spore size reduction was measured in microscope keeping 100 mm scale constant by taking the spore Area (µm^2^) and performed Fisher LSD test and p value was significant (p < = 0.05) (Fig. 5 supplementary).

## Discussion

The marine environment is a rich source of many biological molecules which remain to be underexplored, one such biomolecule are chitinases. In the present study *Alcaligenes faecaliss* was identified based on the colloidal chitin agar based plate assay where out of 30 isolates screened for the presence of the halo zone, the present organism gave the highest activity. In another study conducted by Han et al., 63 chitinase producing bacteria were selected on a colloidal chitin plate based on the clear halo zone and later these chitinolytic bacteria were identified with16S rRNA universal primers (Han et al. 2014). Similar findings were also reported for *Alcaligenes faecalis* AU02 (Annamalai et al. 2011), from seafood industry effluents, which showed enhanced growth and activity in basal media. Additionally, the bacterium *A. ammoinoxydans*, isolated from shrimp shells in the marine environment of Egypt, displayed a distinct halo zone on chitin agar plates. 16 S rRNA sequencing analysis revealed a 97% similarity to *Alcaligenes* s.(Ibrahim et al. 2023).

In the conventional route of purification of *Af* Chi, the end yield was 41.3% with a purification fold of 4.7. In a similar study, conducted on *Paenibacillus pasadenensis* with purification steps involving ammonium sulphate precipitation followed by HiTrap DEAE FF column reported to have an yield of 15.7% and purification fold of 5.30 (Guo et al. 2017). Another study reported 3.26% yield and total purification of 7.45 fold of the chitinase purified from *Streptomyces violaceusniger* (Nagpure and Gupta 2013).

In general, the molecular weight of chitinase can vary ranging from 20 kDa to 90 kDa (Juárez-Hernández et al. 2019; Kim et al. 2021) A thermostable chitinase purified from *Bacillus licheniformans* reported a single band of 67 kDa confirmed by SDS-PAGE and zymogram using 0.1% glycol chitin as substrate (Toharisman et al. 2005). In another study on *Streptomyces violaceusniger* the molecular weight was reported as 56.5 kDa (Nagpure and Gupta 2013). A study conducted on *Bacillus safensis* reported a single band of 58 kDa (Pandya and Saraf 2015). To increase the activity of the purified enzymes, research groups optimize the enzymatic conditions with parameters of pH and temperature. *Af* Chi reported a pH optimum of 7 and temperature maxima of 50 °C. In a similar experiment conducted on *Aspergillus terreus*, the optimum chitinase activity was observed at 50 °C and the pH range between 5 and 7 (Farag et al. 2016). Another strain of *A. faecalis* AU02 showed optimum activity at temperature of 40 °C and pH 8 (Annamalai et al. 2011). Another marine *Bacillus* reported an optimum activity at 37 °C and the enzyme was active between pH 6 to 9 with highest activity at pH 6 (Govindaraj et al. 2024). The thermal stability is another important aspect of enzymes especially if the applications are of industrial nature. *Af* Chi was found to be stable for 24 h at 50 °C. In another study conducted on *A. faecalis* AU02, the enzyme was found atable at 60 °C (Annamalai et al. 2011). Chitinase from *Aspergillus terreus* found to retain 42% activity at 70 °C (Farag et al. 2016). Another chitinase from *Bacillus cereus*, reported to be active in the range of 25 to 55 °C and retained 50% of activity at 60 °C (Wang et al. 2009). As chitinases are metalloenzymes, the impact of the inclusion of the metal co-factors was studied. The activity was found to be enhanced by the inclusion of Na^+^ for *Af* Chi. In another study conducted A study conducted on *Bacillus* the inclusion of Mg^2+^ and Ni^2+^ enhanced the activity while Mn^2+^ and Co^2+^ acted as strong inhibitors (Yuli et al. 2004) reported. After optimizing the process parameters, the kinetic parameters were studied for *Af* Chi which yielded a V_max_ of 17.45 U/mL and K_m_ was 0.187 mg/mL. Beltagy et al. (2018) reported a K_m_ of 0.18 g/mL and a V_max_ of 694.4 U/L of protein for a halophilic chitinase from *A. flavus* (Beltagy et al. 2018). In another study alkaline chitinase from *C. freundii*,* a* K_m_ of 6.77 m/mL and a V_max_ of 2.04 mmol/mL·h were observed (Meruvu and Donthireddy 2014).

Many synthetic fungicides are available to treat the anthracnose disease however, their use in limited due to the regulatory implications, additionally these can have negative effects on the environment, with regard to this. Chitinase is an eco-friendly biological molecule that acts as a bio-fungicide by breaking down the cell walls of fungi, effectively managing fungal infections in plants. Its eco-friendliness and adaptability to various environmental conditions make it a valuable tool for sustainable agriculture.A partially purified chitinase obtained from *P.ehimensis* reported 87% inhibition of spore germination in *C. gloeosporioides* (Seo et al. 2016),*.*whereas the current study, showed 100% inhibition by purified chitinase. In another study, purified and crude chitinase from *S.maltophilia* showed antifungal effects on fungal phytopathogens (Seo et al. 2016). In another study, the crude and partially purified chitinase showed antifungal effects against *A.niger* and *F.solani* mycelium growth (Seo et al. 2016). In conclusion, the purified chitinase (*Af* Chi) could also be explored for treatment of various other fungal diseases found in plants In addition, the chitin oligosaccharides derived from chitinase (*Af* Chi) which has many more applications in immunomodulation, antifungal and anticancer applications as a futuristic goal.

## Electronic supplementary material

Below is the link to the electronic supplementary material.

Supplementary Material 1

## Data Availability

All data generated or analysed will be available on request.

## References

[CR1] Aam BB, Heggset EB, Norberg AL, Sørlie M, Vårum KM, Eijsink VGH (2010) Production of chitooligosaccharides and their potential applications in medicine. Mar Drugs 8: 1482–1517. 10.3390/md8051482 20559485 10.3390/md8051482 PMC2885077

[CR2] Ajuna HB, Lim HI, Moon JH, Won SJ, Choub V, Choi SI, Yun JY, Ahn YS (2023) The prospect of hydrolytic enzymes from *Bacillus* species in the biological control of pests and diseases in forest and fruit tree production. Int J Mol Sci 24. 10.3390/ijms242316889 10.3390/ijms242316889 PMC10707167 38069212

[CR3] Ali AM, Abdel-Rahman TMA, Farahat MG (2024) Bioprospecting of culturable halophilic bacteria isolated from mediterranean solar saltern for extracellular halotolerant enzymes. Microbiol Biotechnol Lett 52:76–87. 10.48022/mbl.2401.01010

[CR4] Anas A, Nilayangod C, Jasmin C, Vinothkumar S, Parameswaran PS, Nair S (2016) Diversity and bioactive potentials of culturable heterotrophic bacteria from the surficial sediments of the Arabian Sea. 3 Biotech 6:1–8. 10.1007/s13205-016-0556-x 10.1007/s13205-016-0556-x PMC5106401 28330310

[CR5] Annamalai N, Rajeswari MV, Vijayalakshmi S, Balasubramanian T (2011) Purification and characterization of chitinase from *Alcaligenes faecalis* AU02 by utilizing marine wastes and its antioxidant activity. Ann Microbiol 61:801–807. 10.1007/s13213-011-0198-5 22131949 10.1007/s13213-011-0198-5 PMC3213332

[CR6] Beltagy EA, Rawway M, Abdul-Raouf UM, Elshenawy MA, Kelany MS (2018) Purification and characterization of theromohalophilic chitinase producing by halophilic *aspergillus flavus* isolated from Suez Gulf. Egypt J Aquat Res 44:227–232. 10.1016/j.ejar.2018.08.002

[CR7] Beygmoradi A, Homaei A (2017) Marine microbes as a valuable resource for brand new industrial biocatalysts. Biocatal Agric Biotechnol 11:131–152. 10.1016/j.bcab.2017.06.013

[CR8] Bhattacharya D, Nagpure A, Gupta RK (2007) Bacterial chitinases: properties and potential. Crit Rev Biotechnol 27:21–28. 10.1080/07388550601168223 17364687 10.1080/07388550601168223

[CR9] Chang WT, Chen CS, Wang SL (2003) An antifungal chitinase produced by *Bacillus cereus* with shrimp and crab shell powder as a carbon source. Curr Microbiol 47:102–108. 10.1007/s00284-002-3955-7 14506855 10.1007/s00284-002-3955-7

[CR10] Daoud L, Ben Ali M (2020) Halophilic microorganisms: interesting group of extremophiles with important applications in biotechnology and environment. INC

[CR11] DasSarma S, DasSarma P (2015) Halophiles and their enzymes: negativity put to good use. Curr Opin Microbiol 25:120–126. 10.1016/j.mib.2015.05.009 26066288 10.1016/j.mib.2015.05.009 PMC4729366

[CR12] Delgado-García M, Valdivia-Urdiales B, Aguilar-González CN, Contreras-Esquivel JC, Rodríguez-Herrera R (2012) Halophilic hydrolases as a new tool for the biotechnological industries. J Sci Food Agric 92:2575–2580. 10.1002/jsfa.5860 22926924 10.1002/jsfa.5860

[CR13] Enamala MK, Chavali M, Pamanji SR, Tangellapally A, Dixit R, Singh M, Kuppam C (2021) Handbook of greener synthesis of nanomaterials and compounds. 10.1016/c2019-0-04948-5. Handb Greener Synth Nanomater Compd

[CR14] Fang H, Liu X, Dong Y, Feng S, Zhou R, Wang C, Ma X, Liu J, Yang KQ (2021) Transcriptome and proteome analysis of walnut (*Juglans regia* L.) fruit in response to infection by *Colletotrichum gloeosporioides*. BMC Plant Biol 21:1–16. 10.1186/s12870-021-03042-1 34059002 10.1186/s12870-021-03042-1 PMC8166054

[CR15] Farag AM, Abd-Elnabey HM, Ibrahim HAH, El-Shenawy M (2016) Purification, characterization and antimicrobial activity of chitinase from marine-derived *aspergillus terreus*. Egypt J Aquat Res 42:185–192. 10.1016/j.ejar.2016.04.004

[CR16] Ferrari AR, Gaber Y, Fraaije MW (2014) A fast, sensitive and easy colorimetric assay for chitinase and cellulase activity detection. Biotechnol Biofuels 7:2–9. 10.1186/1754-6834-7-37 24612932 10.1186/1754-6834-7-37 PMC3975300

[CR17] Fu X, Guo Y, Jin Y, Ma M (2020) Bioconversion of chitin waste using a cold-adapted chitinase to produce chitin oligosaccharides. Lwt 133:109863. 10.1016/j.lwt.2020.109863

[CR18] Gálvez Marroquín LA, Martinez Bolaños M, Cruz Chávez MA, Ariza Flores R, Cruz López JA, Magaña Lira N, de la Cruz LL, Ariza Hernández FJ (2022) Inhibition of mycelial growth and conidium germination of *Colletotrichum* sp. for organic and inorganic products. Agro Prod 15:25–32. 10.32854/agrop.v15i2.2051

[CR19] Ghattavi S, Homaei A (2023) Marine enzymes: classification and application in various industries. Int J Biol Macromol 230:123136. 10.1016/j.ijbiomac.2023.123136 36621739 10.1016/j.ijbiomac.2023.123136

[CR21] Gomaa EZ (2012) Chitinase production by Bacillus thuringiensis and *Bacillus licheniformis*: their potential in antifungal biocontrol. J Microbiol 50:103–111. 10.1007/s12275-012-1343-y 22367944 10.1007/s12275-012-1343-y

[CR20] Gomaa EZ (2021) Microbial chitinases: properties, enhancement and potential applications. Protoplasma 258:695–710. 10.1007/s00709-021-01612-6 33483852 10.1007/s00709-021-01612-6

[CR22] Govindaraj V, Kim SK, Raval R, Raval K (2024) Marine *Bacillus haynesii* chitinase: purification, characterization and antifungal potential for sustainable chitin bioconversion. Carbohydr Res 541:109170. 10.1016/j.carres.2024.109170 38830279 10.1016/j.carres.2024.109170

[CR23] Guo X, Xu P, Zong M, Lou W (2017) Purification and characterization of alkaline chitinase from *Paenibacillus pasadenensis* CS0611. Cuihua Xuebao/Chinese J Catal 38:665–672. 10.1016/S1872-2067(17)62787-6

[CR24] Gurav R, Tang J, Jadhav J (2017) Novel chitinase producer *Bacillus pumilus* RST25 isolated from the shellfish processing industry revealed antifungal potential against phyto-pathogens. Int Biodeterior Biodegrad 125:228–234. 10.1016/j.ibiod.2017.09.015

[CR25] Han K, Il, Patnaik BB, Kim YH, Kwon HJ, Han YS, Han MD (2014) Isolation and characterization of chitinase-producing *Bacillus* and *Paenibacillus* strains from salted and fermented shrimp, Acetes japonicus. J Food Sci 79:665–674. 10.1111/1750-3841.12387 10.1111/1750-3841.12387 24611959

[CR26] Hartl L, Zach S, Seidl-Seiboth V (2012) Fungal chitinases: diversity, mechanistic properties and biotechnological potential. Appl Microbiol Biotechnol 93:533–543. 10.1007/s00253-011-3723-3 22134638 10.1007/s00253-011-3723-3 PMC3257436

[CR27] Horn SJ, Eijsink VGH (2004) A reliable reducing end assay for chito-oligosaccharides. Carbohydr Polym 56:35–39. 10.1016/j.carbpol.2003.11.011

[CR28] Ibrahim M, Selim S, Elhariri M, Farghali HA, Kamel S, Elhelw R (2023) New Chitinolytic *Alcaligenes* species strains isolated from shrimp shells. Egypt J Aquat Biol Fish 27:1191–1205. 10.21608/ejabf.2023.323781

[CR29] Ilham Z, Abdellah H, Wifak B, Mohammed I, Saad I (2013) A novel *Alcaligenes faecalis* antibacterial-producing strain isolated from a Moroccan tannery waste. Afr J Microbiol Res 7:5314–5323. 10.5897/ajmr2013.6029

[CR30] Jenny F, Sultana N, Islam MM, Bhuiyan MMK, B. MA (2019) A review on anthracnose of mango caused by *Colletotrichum* a review on anthracnose of mango caused by *Colletotrichum*. Bangladesh J Plant Pathol 35(12):65–74

[CR31] Juárez-Hernández EO, Casados-Vázquez LE, Brieba LG, Torres-Larios A, Jimenez-Sandoval P, Barboza-Corona JE (2019) The crystal structure of the chitinase ChiA74 of *Bacillus thuringiensis* has a multidomain assembly. Sci Rep 9:1–10. 10.1038/s41598-019-39464-z 30796308 10.1038/s41598-019-39464-z PMC6385353

[CR32] Karan R, Kumar S, Sinha R, Khare SK (2012) Halophilic microorganisms as sources of novel enzymes bt—microorganisms in sustainable agriculture and biotechnology. Springer Netherlands, Dordrecht

[CR33] Karthik N, Binod P, Pandey A (2015) Purification and characterisation of an acidic and antifungal chitinase produced by a *Streptomyces* Sp. Bioresour Technol 188:195–201. 10.1016/j.biortech.2015.03.006 25824594 10.1016/j.biortech.2015.03.006

[CR34] Kim SK, Park JE, Oh JM, Kim H (2021) Molecular characterization of four alkaline chitinases from three chitinolytic bacteria isolated from a mudflat. Int J Mol Sci 22. 10.3390/ijms222312822 10.3390/ijms222312822 PMC8658002 34884628

[CR35] Kotb E, Alabdalall AH, Alghamdi AI, Ababutain IM, Aldakeel SA, Al-Zuwaid SK, Algarudi BM, Algarudi SM, Ahmed AA, Albarrag AM (2023) Screening for chitin degrading bacteria in the environment of Saudi Arabia and characterization of the most potent chitinase from *Streptomyces variabilis* Am1. Sci Rep 13:1–12. 10.1038/s41598-023-38876-2 37474592 10.1038/s41598-023-38876-2 PMC10359409

[CR36] Kumar M, Brar A, Vivekanand V, Pareek N (2018) Process optimization, purification and characterization of a novel acidic, thermostable chitinase from *Humicola Grisea*. Int J Biol Macromol 116:931–938. 10.1016/j.ijbiomac.2018.05.125 29782982 10.1016/j.ijbiomac.2018.05.125

[CR37] Kurniawan E, Leelakriangsak M, Panphon S (2022) Antifungal chitinase production by *Bacillus paramycoides* B26 using Squid Pen Powder as a Carbon source. J Pure Appl Microbiol 16:2496–2506. 10.22207/JPAM.16.4.09

[CR38] Liu C, Shen N, Wu J, Jiang M, Shi S, Wang J, Wei Y, Yang L (2020) Cloning, expression and characterization of a chitinase from *Paenibacillus chitinolyticus* strain UMBR 0002. PeerJ 2020:1–23. 10.7717/peerj.8964 10.7717/peerj.8964 PMC7207210 32411515

[CR39] Mander P, Cho SS, Choi YH, Panthi S, Choi YS, Kim HM, Yoo JC (2016) Purification and characterization of chitinase showing antifungal and biodegradation properties obtained from *Streptomyces anulatus* CS242. Arch Pharm Res 39:878–886. 10.1007/s12272-016-0747-3 27215829 10.1007/s12272-016-0747-3

[CR40] Meruvu H, Donthireddy SRR (2014) Purification and characterization of an antifungal chitinase from *Citrobacter freundii* str. nov. haritD11. Appl Biochem Biotechnol 172:196–205. 10.1007/s12010-013-0540-4 24065288 10.1007/s12010-013-0540-4

[CR41] Moreno M, de L, Pérez D, García MT, Mellado E (2013) Halophilic bacteria as a source of novel hydrolytic enzymes. Life 3:38–51. 10.3390/life3010038 25371331 10.3390/life3010038 PMC4187191

[CR42] Muthukumar A (2018) Field evaluation of new fungicide molecule (Ridomil gold 68% wp) against leaf spot of Chilli. Met Ala Alaxyl Xyl-M

[CR43] Nagpure A, Gupta RK (2013) Purification and characterization of an extracellular chitinase from antagonistic *Streptomyces violaceusniger*. J Basic Microbiol 53:429–439. 10.1002/jobm.201100648 22915152 10.1002/jobm.201100648

[CR44] Number C (1999) SyproOrange Biorad. 1–11

[CR45] Pandya U, Saraf M (2015) Purification and characterization of antifungal chitinase from *Bacillus safensis* MBCU6 and its application for production of chito-oligosaccharides. Biol 70:863–868. 10.1515/biolog-2015-0112

[CR46] Paulsen SS, Andersen B, Gram L, MacHado H (2016) Biological potential of chitinolytic marine bacteria. Mar Drugs 14:1–17. 10.3390/md14120230 10.3390/md14120230 PMC5192467 27999269

[CR47] Pawaskar GM, Raval K, Rohit P, Shenoy RP, Raval R (2021) Cloning, expression, purification and characterization of chitin deacetylase extremozyme from halophilic *Bacillus aryabhattai* B8W22. 3 Biotech 11:1–13. 10.1007/s13205-021-03073-3 10.1007/s13205-021-03073-3 PMC8636556 34917446

[CR48] Peralta-Ruiz Y, Rossi C, Grande-Tovar CD, Chaves-López C (2023) Green management of postharvest anthracnose caused by *Colletotrichum gloeosporioides*. J Fungi 9. 10.3390/jof9060623 10.3390/jof9060623 PMC10302910 37367558

[CR49] Purwanto MGM (2016) The role and efficiency of ammonium sulphate precipitation in purification process of papain crude extract. Procedia Chem 18:127–131. 10.1016/j.proche.2016.01.020

[CR50] Qiu J, Han R, Wang C (2021) Microbial halophilic lipases: a review. J Basic Microbiol 61:594–602. 10.1002/jobm.202100107 34096085 10.1002/jobm.202100107

[CR51] Rathore AS, Gupta RD (2015) Chitinases from bacteria to human: properties, applications, and future perspectives. 2015:1–8. 10.1155/2015/791907 10.1155/2015/791907 PMC4668315 26664744

[CR52] Sandhu JS, Sidhu MK, Yadav IS (2017) Control of fungal diseases in agricultural crops by chitinase and glucanase transgenes

[CR53] Sasi A, Duraipandiyan N, Marikani K, Dhanasekaran S, Al-Dayan N, Venugopal D (2020) Identification and characterization of a newly isolated chitinase-producing strain *Bacillus licheniformis* SSCL-10 for chitin degradation. Archaea 2020. 10.1155/2020/8844811 10.1155/2020/8844811 PMC7669355 33223963

[CR54] Senol M, Nadaroglu H, Dikbas N, Kotan R (2014) Purification of chitinase enzymes from *Bacillus subtilis* bacteria TV-125, investigation of kinetic properties and antifungal activity against *Fusarium Culmorum*. Ann Clin Microbiol Antimicrob 13:1–7. 10.1186/s12941-014-0035-3 25112904 10.1186/s12941-014-0035-3 PMC4236515

[CR55] Seo DJ, Lee YS, Kim KY, Jung WJ (2016) Antifungal activity of chitinase obtained from *Paenibacillus ehimensis* MA2012 against conidial of *Collectotrichum gloeosporioides* in vitro. Microb Pathog 96:10–14. 10.1016/j.micpath.2016.04.016 27133265 10.1016/j.micpath.2016.04.016

[CR56] Sharma S, Kumar S, Khajuria A, Ohri P, Kaur R, Kaur R (2020) Biocontrol potential of chitinases produced by newly isolated *Chitinophaga* sp. S167. World J Microbiol Biotechnol 36:1–15. 10.1007/s11274-020-02864-9 10.1007/s11274-020-02864-9 32524202

[CR57] Sonawane KD, Dandagal NR, Naikwadi AG, Gurav PT, Anapat SV, Nadaf NH, Jadhav DB, Waghmare SR (2016) Intergeneric fusant development using chitinase preparation of *Rhizopus Stolonifer* NCIM 880. 10.1186/s13568-016-0287-8. AMB Express 6 10.1186/s13568-016-0287-8 PMC5108735 27844458

[CR58] Souza CP, Almeida BC, Colwell RR, Rivera ING (2011) The importance of chitin in the marine environment. Mar Biotechnol 13:823–830. 10.1007/s10126-011-9388-1 10.1007/s10126-011-9388-1 21607543

[CR59] Subramanian K, Balaraman D, Panangal M, Nageswara Rao T, Perumal E, Amutha R, Kumarappan A, Sampath Renuga P, Arumugam S, Thirunavukkarasu R, Aruni W, Yousef AlOmar S (2022) Bioconversion of chitin waste through *Stenotrophomonas maltophilia* for production of chitin derivatives as a Seabass enrichment diet. Sci Rep 12:1–11. 10.1038/s41598-022-08371-1 35314727 10.1038/s41598-022-08371-1 PMC8938544

[CR60] Toharisman A, Suhartono MT, Spindler-Barth M, Hwang JK, Pyun YR (2005) Purification and characterization of a thermostable chitinase from *Bacillus licheniformis* Mb-2. World J Microbiol Biotechnol 21:733–738. 10.1007/s11274-004-4797-1

[CR61] Trudel J, Asselin A (1990) Detection of chitin deacetylase activity after polyacrylamide gel electrophoresis. Anal Biochem 189:249–253. 10.1016/0003-2697(90)90116-Q 2281870 10.1016/0003-2697(90)90116-q

[CR62] Wang SL, Chao CH, Liang TW, Chen CC (2009) Purification and characterization of protease and chitinase from *Bacillus cereus* TKU006 and conversion of marine wastes by these enzymes. Mar Biotechnol 11:334–344. 10.1007/s10126-008-9149-y 10.1007/s10126-008-9149-y 18843519

[CR63] Yan Q, Fong SS (2015) Bacterial chitinase: nature and perspectives for sustainable bioproduction. Bioresour Bioprocess 2. 10.1186/s40643-015-0057-5

[CR64] Ye H, Wang Q, Zhu F, Feng G, Yan C, Zhang J (2020) Antifungal activity of alpha-mangostin against including. Molecules 25:1–14 10.3390/molecules25225335 PMC7696833 33207599

[CR65] Yuli PE, Suhartono MT, Rukayadi Y, Hwang JK, Pyun YR (2004) Characteristics of thermostable chitinase enzymes from the Indonesian *Bacillus* sp.13.26. Enzyme Microb Technol 35:147–153. 10.1016/j.enzmictec.2004.03.017

[CR66] Zhang C, Kim SK (2010) Research and application of marine microbial enzymes: Status and prospects. Mar Drugs 8:1920–1934. 10.3390/md8061920 20631875 10.3390/md8061920 PMC2901830

[CR67] Zhang W, Ma J, Yan Q, Jiang Z, Yang S (2021) Biochemical characterization of a novel acidic chitinase with antifungal activity from *Paenibacillus xylanexedens* Z2–4. Int J Biol Macromol 182:1528–1536. 10.1016/j.ijbiomac.2021.05.111 34022308 10.1016/j.ijbiomac.2021.05.111

