# Supplementary material for: Marine chitinase AfChi: green defense management against Colletotrichum gloeosporioides and anthracnose
Source: AMB Express. 2024 Nov 20;14:128. doi: 10.1186/s13568-024-01786-1 (PMC11579262; doi:10.1186/s13568-024-01786-1)
Supplement: Supplementary file 1 — Supplementary Material 1 [file 13568_2024_1786_MOESM1_ESM.docx]

**Marine chitinase *Af*Chi: Green defense management against *Colletotrichum gloeosporioides* and anthracnose**

**Rajesh KM^1^, Keyur Raval^2*^, Ritu Raval^1*^**

^1^ Department of Biotechnology, Manipal Institute of Technology, Manipal Academy of Higher Education, Manipal - 576104, Karnataka, India.

^2^Department of Chemical Engineering, National Institute of Technology, Surathkal, Mangalore – 575025, Karnataka, India.

*Corresponding authors:

keyurnraval@nitk.edu.in, Tel: +91-824-2473637 (KR)

ritu.raval@manipal.edu, Tel: +91-9019062782 (RR)

**Applied Microbiology and Biotechnology**


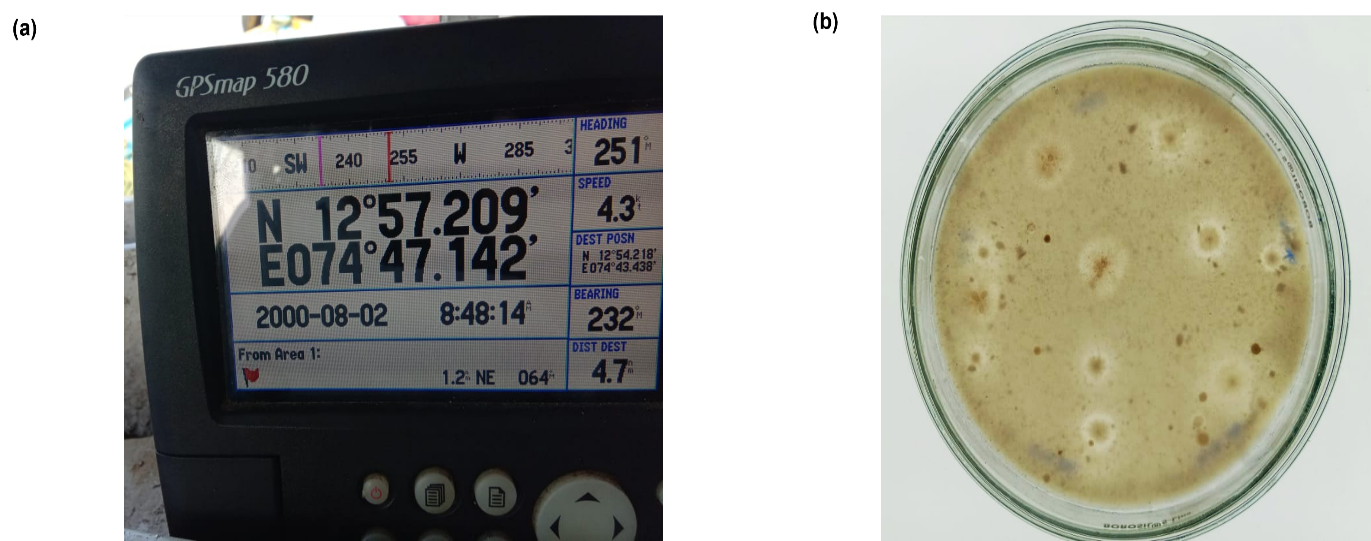


**Fig.1** Isolation of bacteria from marine sediment of Arabian Sea a) Collection site location. b) Bacteria exhibiting chitinolytic activity on semi-synthetic colloidal chitin agar (SSCA) plates.


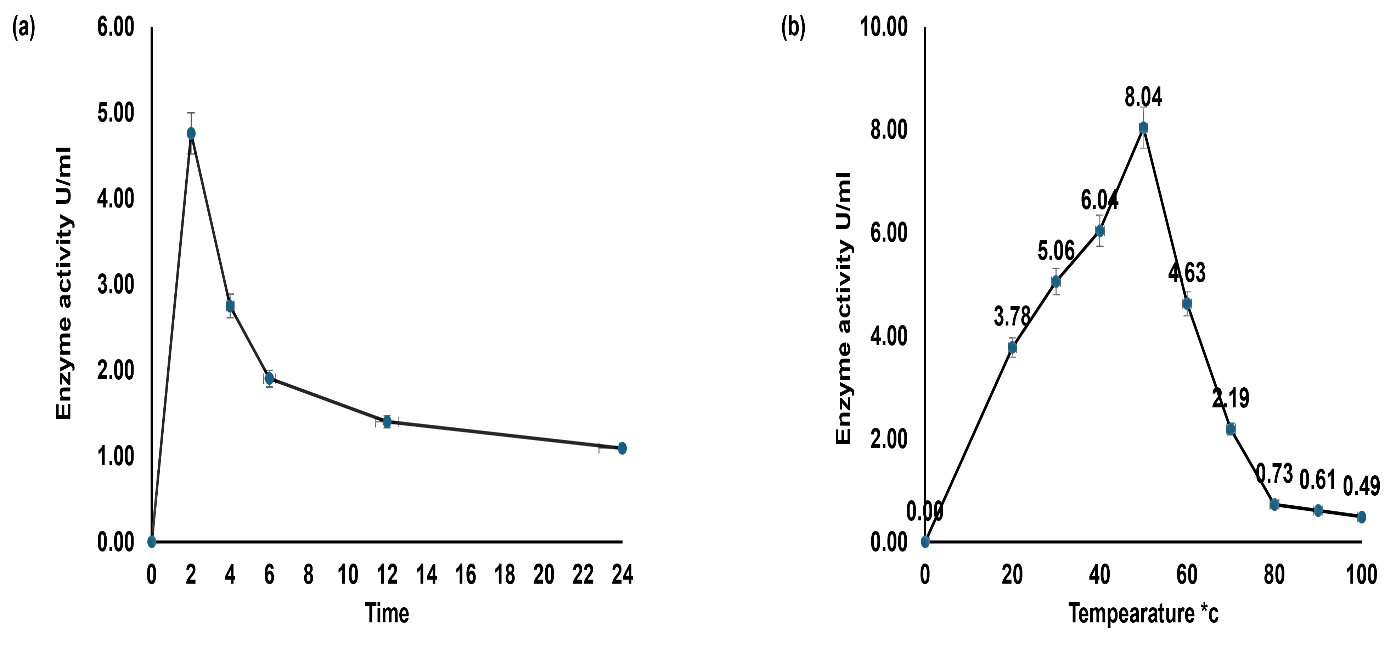


**Fig. 2** a) Effect of purified chitinase activity against different time intervals in constant 50°C. b) Effect of purified chitinase enzyme stability after 24hr incubation in room temperature with different temperature (20-100°C).

**
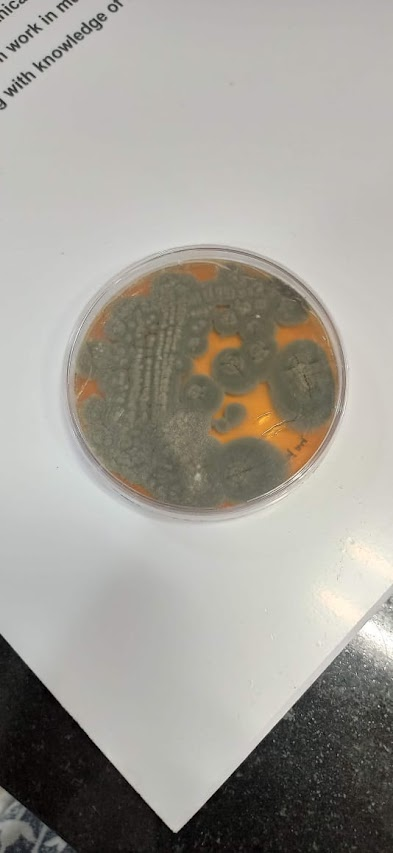
**

**Fig. 3** *Colletotrichum gloeosporioides* exhibiting typical morphological characteristics

**Fig. 4** Conidiospore germination inhibition percentage was calculated ordinary one-way ANOVA test done between 1) control, 2) Purified chitinase, 3) Standard and obtained significant result *p<= 0.05.





**Fig. 5** Fisher LSD test image of size reduction of conidiospores in a) control, b) Enzyme treated, c) Standard.
